# Signaling Through the Erythropoietin Receptor Affects Angiogenesis in Retinovascular Disease

**DOI:** 10.1167/iovs.61.10.23

**Published:** 2020-08-12

**Authors:** Colin A. Bretz, Aniket Ramshekar, Eric Kunz, Haibo Wang, M. Elizabeth Hartnett

**Affiliations:** John A. Moran Eye Center, University of Utah, Salt Lake City, Utah, United States

**Keywords:** erythropoietin, erythropoietin receptor, oxygen induced retinopathy, neovascularization, angiogenesis

## Abstract

**Purpose:**

Exogenous erythropoietin (EPO) is being considered for tissue protection and angiogenesis in retinal vascular diseases. However, studies are limited by insufficient tools to address signaling effects through the EPO receptor (EPOR). We used a humanized mouse model of hypoactive EPOR signaling to test the hypothesis that EPOR signaling supports angiogenesis in retinovascular diseases.

**Methods:**

Humanized Knockin EPOR mice (hWt*EPOR*) with hypoactive EPOR signaling were compared to littermate wild-type mice (WT). Postnatal day (p)7 mice of each genotype were exposed to 75% oxygen for five days, followed by 21% oxygen in the oxygen-induced retinopathy model (OIR) and compared to room-air (RA)–raised pups. At time points after OIR, pups were sacrificed, and flat-mounted, lectin-stained retinas were analyzed for central avascular area or intravitreal neovascular area (IVNV). Flash-frozen retinas were analyzed for angiogenic protein (Epo, VEGF, p-Stat3) and gene (*Vegfa*, *Kdr*, *Epo*, *Hif1α*, *Hif2α*) expression levels.

**Results:**

In OIR, hWt*EPOR* mice had increased AVA compared with WT at p8, p12, and p17, but there was no difference in IVNV between hWt*EPOR* and WT mice at p17. Although VEGF and p-STAT3 proteins were increased in WT at p17 OIR, there were no differences in retinal angiogenic factor expression levels between hWt*EPOR* and WT OIR at p17 despite similar areas of IVNV.

**Conclusions:**

Our data support the hypothesis that EPOR signaling was associated with regrowth of vascularization following oxygen-induced capillary dropout and played a role in intravitreal angiogenesis. Additional study of EPOR signaling regulation on other angiogenic factor pathways may be considered.

Experimental evidence supports erythropoietin (EPO) as a cytoprotective agent for endothelial cells of newly formed capillaries acutely exposed to high oxygen,^1^ and to retinal neurons after oxygen-induced vascular dropout and remodeling.^2^ Studies also show EPO is produced in tissues besides the kidney, including in retina,^1^^,^^3^ and that its receptor is expressed in the retina and choroid.^4^^–^^6^ Based on the benefit from experimental studies, exogenously delivered EPO has been proposed in clinical trials for neuroprotection in retinal neural and vascular diseases,^7^^–^^10^ including diabetic retinopathy and retinopathy of prematurity (ROP).^11^^,^^12^ However, there have been discrepancies in outcomes between different studies in the clinical literature, which may be due in part to the difficulty determining which receptors exogenously delivered EPO triggers signaling through in tissues of experimental interest.

EPO binds its receptor EPO receptor (EPOR) to trigger signaling through the JAK/STAT5 signaling pathway for its most known function of erythropoiesis.^13^ EPO is also believed to bind other receptors to be tissue protective,^14^^–^^16^ and there is evidence of EPOR interacting with VEGF receptor 2 to cause angiogenesis.^17^ Most studies have been limited, because antibodies to EPOR are unreliable for immunohistochemistry,^18^^,^^19^ so it is unclear whether administered EPO activates signaling processes. In addition, EPO or EPOR knockout mice are lethal in embryonic life, limiting the study of tissue protection in adults.^20^ To address these limitations, we used humanized transgenic mice in which the murine *EpoR* gene was replaced with the human *EPOR* gene,^21^ resulting in hypoactive signaling through human EPOR because of reduced transmembrane protein stability and not from reduced EPO/EPOR binding.^22^^,^^23^

After vascular dropout and regrowth from high oxygen-induced retinopathy (OIR), we previously reported that adult mice with normal EPOR signaling compared with those with hypoactive EPOR signaling had partial restoration of connectivity between rods and the inner nuclear layer through ectopic rod bipolar cell neurites that resulted in improved b-wave amplitudes in the electroretinograms.^2^ Although there were differences in neural structure throughout the retina between the transgenic mice with hypoactive EPOR signaling and littermate wild-type mice after OIR, there were also differences between the central retina where vascular dropout had occurred and the peripheral retina in the transgenic mice, thus raising the question whether some differences seen were related to vascular effects on the retina and not directly on EPOR-induced effects on neural processing.

Previous experimental studies using the murine OIR model show that EPO given early to mice reduces vaso-obliteration of newly formed capillaries from high oxygen but does not increase later pathologic intravitreal angiogenesis, whereas EPO administered later does not affect either area of vaso-obliteration or pathologic intravitreal angiogenesis.^1^ However, in another OIR study, EPO knockdown achieved with intravitreal siRNA to EPO reduces pathologic intravitreal angiogenesis but also allows regrowth of intraretinal angiogenesis.^4^ These studies suggest that EPO is protective in high oxygen but also raises the question whether the EPO effect is partly through angiogenesis. The investigators bring up the problem of not having tools available to test the role of EPOR signaling in the retina.

To address the role of EPOR signaling as opposed to EPO ligand effects, we tested the hypothesis that signaling through EPOR was angiogenic after vascular loss by exposing humanized transgenic mice with hypoactive EPOR signaling and wild-type littermates with normal EPOR signaling to OIR as a model of vascular remodeling. This model is analogous to retinovascular diseases, such as diabetic retinopathy, retinal vein occlusion and some aspects of ROP.

## Methods

### Humanized Mouse Model

All procedures were conducted in accordance with University of Utah (Guide for the Care and Use of Laboratory Animals) and the Association for Research in Vision and Ophthalmology Statement for the Use of Animals in Ophthalmic and Vision Research, and were approved by the Institutional Animal Care and Use Committee (IACUC) and the Institutional Biosafety Committee of the University of Utah.

These studies were conducted using humanized knock-in mice in which the human *EPOR* gene was knocked into the endogenous mouse *EpoR* locus, via gene targeting, and then backcrossed to a C57BL/6 background.^21^ Subsequent generations were maintained on a C57BL/6 background and routinely tested for *Rd1, Rd8 and Gnat2* mutations.^24^ For all studies, heterozygous mWt*EpoR*/hWt*EPOR* mice were bred to obtain litters that contained heterozygous mWt*EpoR*/hWt*EPOR* mice, homozygous mWt*EpoR*/mWt*EpoR* mice (denoted WT), and homozygous hWt*EPOR*/hWt*EPOR* mice (denoted hWt*EPOR*). This breeding scheme was chosen to produce homozygous WT and hWt*EPOR* littermates for analysis and to control for any maternal effect of the human *EPOR* gene. mRNA analysis of the murine *EpoR* gene confirmed that relative to WT littermates, hWt*EPOR* mice did not express detectable levels of the murine *EpoR* gene whereas heterozygous mice exhibited partial expression (Supplementary Fig. S1). Male and female mice were included in all experiments.

### Oxygen Induced Retinopathy Model

At postnatal day 7 (p7), litters and dams from heterozygous hWt*EPOR*/mWt*EpoR* breeding pairs were placed into an OxyCycler (Biospherix, Parish, NY, USA) and exposed to 75% oxygen for five days.^25^ On postnatal day 12 (p12), litters were removed from the OxyCycler and returned to room air (RA, 21% oxygen). To control for epigenetic effects from exposure to hyperoxia, animals that were exposed to hyperoxia (dam or pup) were not used for additional breeding.

### Tissue Harvest and Flat Mounts

Enucleated eyes were harvested for flat mounts at p3 and p7 to analyze physiological retinal vascular development, and at p8, p12, and p17 to analyze the effects of OIR. Immediately after enucleation, corneas were cut to optimize fixation of the retinas, and eyes were then fixed for one hour in 4% paraformaldehyde (PFA). Once fixed, the cornea was excised and lens gently removed without disrupting the retina while visualizing the eye using a dissecting microscope. The entire retina with intact ora serrata was then gently separated from the underlying sclera. Hyaloidal vessels and the remaining vitreous were carefully removed. The retina was flattened by making four radial incisions. The flattened retinas were then stained with Alexa Fluor 568 conjugated *Griffonia* *simplicifolia* (Bandeiraea) isolectin B4 overnight and mounted on a slide for image capture.

### Quantification of Avascular Area and Neovascularization

Images of the superficial retinal vasculature were captured using an inverted fluorescence microscope (Olympus, Tokyo, Japan). Images of complete retinal flat mounts were created using the scan-slide stitching function of Metamorph imaging software (Molecular Devices, Inc., Sunnyvale, CA, USA). Image analysis was performed by at least two masked observers. Measurements were made using Image J software (National Institutes of Health, Bethesda, MD, USA). The areas of avascular retina and intravitreal neovascularization (IVNV) were calculated as a percentage of total retinal area for each flat mount. At p3, n = 8–18 per group; at p7, n = 6–8 per group; at p8, n = 10–15 per group; at p12, n = 9–15 per group; and at p17, n = 13–17 per group.

### Serum EPO and VEGF Enzyme-Linked Immunosorbent Assay

Blood was collected from RA and OIR WT and hWt*EPOR* mice at p17 into non-heparin-treated centrifuge tubes. Blood was allowed to coagulate for two hours at 4°C and then spun down for 25 minutes at 2000*g* at 4°C. The serum supernatant was collected and assayed as recommended using murine Epo and VEGF enzyme-linked immunosorbent assays (ELISAs; R&D, Minneapolis, MN, USA). Each sample was run in duplicate, and the average concentration was used for analysis. For Epo ELISA, n = 6–9 per group and for VEGF ELISA, n = 4–9 per group.

### Hematocrit

Blood was collected during tissue harvest at all analyzed time points into heparin-treated micro-hematocrit tubes (VWR, Radnor, PA, USA). The tubes were spun for five minutes in a hematocrit microcentrifuge and read using a capillary microhematocrit reader to determine the percentage of packed red blood cells/volume. Tubes were collected in duplicate, and the average value was used for analysis. At p3, n = 8–13 per group; at p7, n = 5–7 per group; at p8, n = 4–17 per group; at p12, n = 9–21 per group; at p14, n = 4–8 per group; and at p17, n = 9–15 per group.

### Western Blot Analysis

Protein lysates were collected from homogenized retinal tissues at p8, p12, p14, and p17 in radio immunoprecipitation assay (RIPA) buffer (20 mmol/L Tris pH 7.4, 120 mmol/L NaCl, 0.5% sodium deoxycholic acid, 1% Triton X-100, 0.1% SDS, 10% glycerol) containing protease inhibitor cocktail (Roche Diagnostics, Indianapolis, IN, USA) and phosphatase inhibitor cocktail (ThermoFisher Scientific, Waltham, MA, USA) on ice. Protein lysates were clarified by centrifugation at 13,000*g* rpm for five minutes at 4°C. Protein concentration in the supernatant was quantified by bicinchoninic acid assay (BCA). Eight micrograms of protein from each treatment group was loaded into NuPAGE 4% to 12% Bis-Tris Gels (Invitrogen, Carlsbad, CA, USA), transferred to a PVDF membrane, and incubated with antibodies to rabbit anti-EPO (1:500, Santa Cruz Biotechnology, Santa Cruz, CA, USA), rabbit anti-VEGF (1:500, Santa Cruz Biotechnology), rabbit anti-phosphorylated STAT3 (Y705, 1:1000; Cell Signaling Technology, Danvers, MA, USA), or mouse anti-STAT3 (1:1000, Cell Signaling Technology) at 4°C overnight. After incubating with primary antibodies, membranes were then probed with HRP conjugated goat antirabbit secondary antibody or goat antimouse secondary antibody (1:3000, ThermoFisher Scientific) at room temperature for 1 hr. All the membranes were then re-probed with HRP conjugated β-actin (1:3000, Santa Cruz Biotechnology) as loading controls. Densitometry analysis was performed on exposed films using FIJI software and normalized to β-actin. Only one eye per mouse was used for analysis. At p8, n = 5–10 per group; p12, n = 6 per group; p14, n = 4–8 per group; and p17, n = 5–10 per group.

### Quantitative Real Time-PCR Analysis

Eyes from RA and OIR WT and hWt*EPOR* mice were harvested at p17. Eyes were enucleated, washed in phosphate buffered saline (Genesee Scientific, San Diego, CA, USA), and then retinas were immediately dissected and flash frozen. For RT-PCR, tissue was placed into buffer RLT and sonicated prior to mRNA isolation using a Qiagen RNEASY kit (Qiagen, Valencia, CA, USA). The cDNA was reverse transcribed from mRNA samples and evaluated using Taqman Gene Expression Assays (ThermoFisher Scientific). The ΔCT values for target genes were calculated using both *Tata-Box Binding Protein* and *β-actin* as house-keeping controls and the 2^−ΔΔCT^ was calculated relative to the RA WT group. Only one eye per mouse was used for analysis. At p14, n = 4–8 per group; and at p17, n = 6–8 per group.

### Statistical Analysis

All statistical analyses were performed using STATA15 software (StataCorp, College Station, TX, USA). For in vivo animal studies, a multivariable mixed effects linear regression model was used to account for variability between litters and individual animals. For analysis of Western blot densitometry and RT-PCR, a Student's *t*-test was used to compare groups, and for RT-PCR, the ΔCT values were used to determine significance. *P* < 0.05 was considered statistically significant.

## Results

### Serum EPO in hWt*EPOR* and WT mice

**Figure 1.**
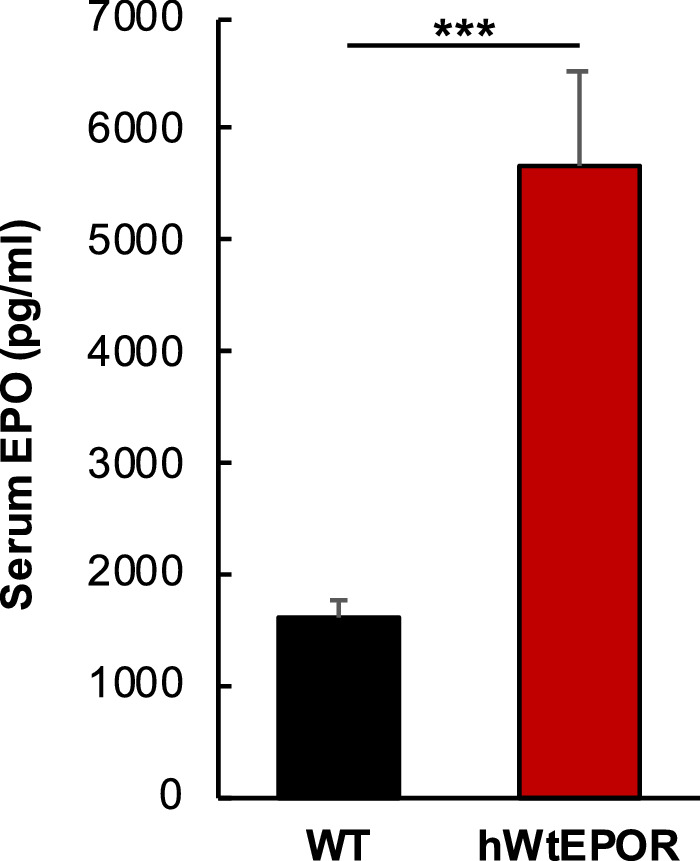
Effect of altered EPOR signaling on serum EPO levels. Comparison of serum EPO levels in WT and hWt*EPOR* mice at p17 (n = 6 for both groups). Results are means ±SEM. *** = *P* < 0.001.

Anemia in extremely premature infants born younger than 28 weeks gestation has been reported in association with high serum EPO and an increased risk of ROP,^26^ but EPO itself was not believed to be the cause; rather anemia was concluded to be the actual risk factor and high EPO was a response to anemia. To determine the relevance of the hWt*EPOR* mice as a model to human disease, we confirmed hWt*EPOR* mice raised in RA have increased serum Epo compared to littermate WT mice (Fig. 1) but had no further increase in Epo at p17 after being exposed to OIR (data not shown). These findings are consistent with findings described in premature infants described above^26^ and, taken together, provide translational support for the use of the hWt*EPOR* model to address our hypothesis.

### Effect of EPOR Signaling on Hematocrit

**Figure 2.**
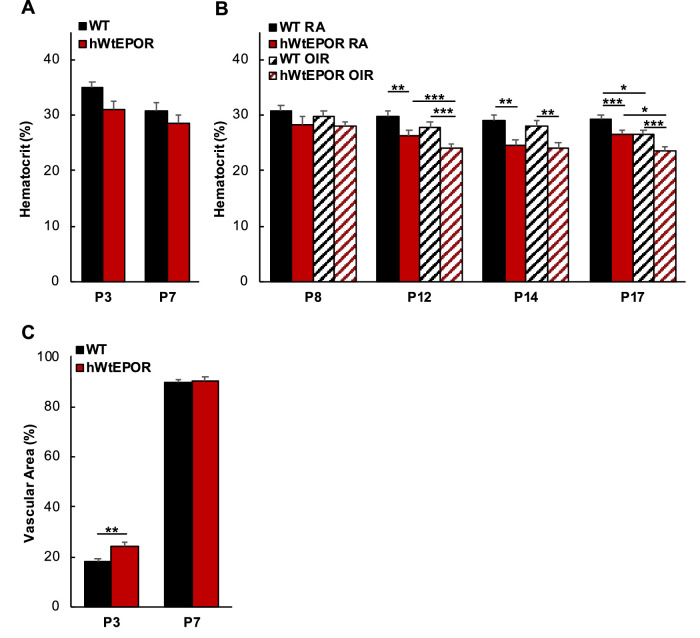
Effect of altered EPOR signaling on hematocrit and vascular development. (**A)** Comparison of hematocrit in WT and hWt*EPOR* mice at p3 and p7 (p3: WT (n = 13), hWt*EPOR* (n = 8*);* p7: WT (n = 8), hWt*EPOR* (n = 7)). (**B)** Comparison of hematocrit in OIR treated and RA control pups at p8, p12, p14, and p17 (p8: WT RA (n = 7), hWt*EPOR* RA (n = 4), WT OIR (16), hWt*EPOR* OIR (17); p12: WT RA (n = 12), hWt*EPOR* RA (n = 10), WT OIR (n = 9), hWt*EPOR* OIR (n = 16); p14: WT RA (n = 6), hWt*EPOR* RA (n = 5), WT OIR (n = 4), hWt*EPOR* OIR (n = 8); p17: WT RA (n = 12), hWt*EPOR* RA (n = 10), WT OIR (n = 15), hWt*EPOR* OIR (n = 9)). (**C)** Comparison of total vascular coverage at p3 and p7 in WT and hWt*EPOR* mice (p3: WT (n = 18), hWt*EPOR* (n = 8); p7: WT (n = 6) and hWt*EPOR* (n = 8)). Results are means ± SEM. *P < 0.05, ** *P* < 0.01, and *** *P* < 0.001.

Because of the association of anemia with high serum EPO in extremely premature infants and because of the known increase in serum Epo in the hWt*EPOR* mice,^26^ we measured hematocrits in WT and hWt*EPOR* mice raised in RA or the OIR model to assess whether hWt*EPOR* mice experienced anemia (Figs. 2A, 2B). At time points p3 and p7 before oxygen exposure (Fig. 2A) and in RA raised mice at p8 (Fig. 2B), there was no significant difference in hematocrits between WT and hWt*EPOR* mice. However, at p12, p14, and p17, hWt*EPOR* mice in RA had significantly reduced hematocrit levels compared to WT in RA (Fig. 2B). After one day of 75% oxygen at p8, there was no difference in hematocrit between WT and hWt*EPOR*, but at p12, hyperoxia was associated with lower hematocrit in hWt*EPOR* mice compared to RA. At p17, exposure to the OIR model was associated with lower hematocrit compared to RA in each genotype. These findings suggested that hypoactive EPOR signaling reduced hematocrit, consistent with the known role of EPO in erythropoiesis, and that the reduced hematocrit associated with high oxygen at p12 and p17 was more pronounced when EPOR signaling was hypoactive. This also related to the findings in the Lundgren study relating hematocrit and ROP in human preterm infants.^26^

### Effect of EPOR Signaling on Physiological Retinal Vascular Development

Our hypothesis predicted that low EPOR signaling would reduce angiogenesis and therefore would lead to reduced retinal vascular development. To test this prediction, we measured vascular area in littermate WT and hWt*EPOR* mice at p3 and p7 (Fig. 2C). The hWt*EPOR* mice with reduced EPOR signaling showed no decrease in vascularization compared with WT mice and in fact had increased vascular area at p3. By p7, there was no difference in retinal vascular areas between genotypes. This catch-up angiogenesis suggests that from p3 to p7 there was increased vascular growth in WT mice compared to hWt*EPOR* littermates. Importantly, it also establishes that genotypes had similar vascular areas at p7 when mice were placed into high oxygen, thus allowing for a clearer interpretation of results between genotypes in OIR.

### Effect of EPOR Signaling on High Oxygen-Induced Vascular Dropout

**Figure 3.**
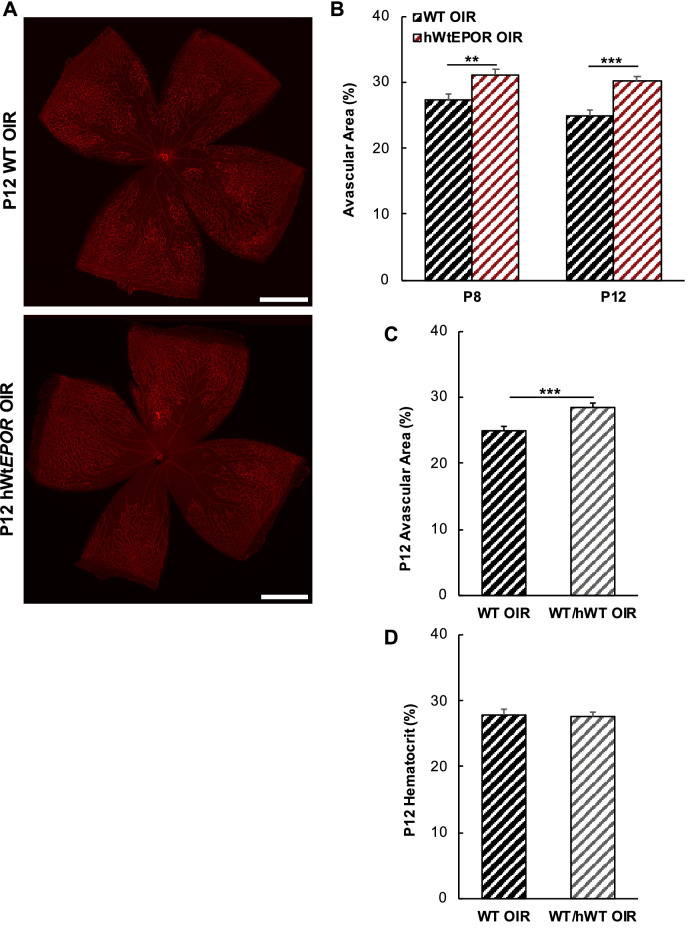
Effect of reduced EPOR signaling on avascular area in OIR at p8 and p12. (**A)** Representative images of retinal flat mounts at p12 from OIR treated WT and hWt*EPOR* mice. (**B)** Comparison of avascular area at p8 and p12 in WT and hWt*EPOR* mice (p8: WT (n = 10), hWt*EPOR* (n = 15); at p12: WT (n = 11), hWt*EPOR* (n = 15)). (**C)** Comparison of central avascular area in WT and heterozygous mice at p12 (WT (n = 11), heterozygous (n = 9)). (**D)** Comparison of hematocrit in WT and heterozygous mice at p12 (WT (n = 9), heterozygous (n = 21)). Results are means ±SEM. Scale bar = 500 µmol/L. ** *P* < 0.01 and *** *P* < 0.001.

Exogenous EPO was previously reported to reduce hyperoxia-induced vascular loss by stabilizing newly developed retinal capillaries from oxygen dropout.^1^ We predicted that the effect of exogenous EPO would be from signaling through EPOR, and that reduced EPOR signaling would increase avascular retina following high oxygen. To test this prediction, we measured central avascular areas at p8 and p12 in WT and hWt*EPOR* mice exposed to 75% oxygen for one and five days (Fig. 3), respectively. In support of the prediction, hWt*EPOR* mice had significantly more central avascular area at both p8 and p12 compared with their respective WT littermate controls (Fig. 3B). There was no difference in the percent of central avascular area/total retinal area between p8 and p12 of mice of the same genotype, suggesting that most vascular dropout occurred within 24 hours of exposure to high oxygen. It was also noted that the increased avascular area from high oxygen at p8 in hWt*EPOR* compared with WT mice occurred when hematocrit levels were similar between the genotypes (Fig. 2B). There was no further increase in avascular retina at p12 even though hematocrit was lower in p12 hWt*EPOR* mice compared with WT mice. Together these findings provide evidence that increased avascular area observed in hWt*EPOR* mice was associated with reduced EPOR signaling and not with differences in hematocrit levels.

We further addressed the hypothesis that EPOR signaling and not hematocrit was important in vascular stability by performing a gene dosage experiment to determine the association between hematocrit and avascular retina in heterozygous and homozygous littermates at p12 (Figs. 3C, 3D). After five days in 75% oxygen, there was increased avascular area in the heterozygous mice compared to littermate WT controls (Fig. 3C), but there was no difference in hematocrit between heterozygous and WT littermates (Fig. 3D). Together, the data provide additional support that EPOR signaling affected retinal vascularity during hyperoxia through non-erythropoietic effects since there was no associated change in hematocrit.

### Effect of EPOR Signaling on Vascular Regrowth After OIR

**Figure 4.**
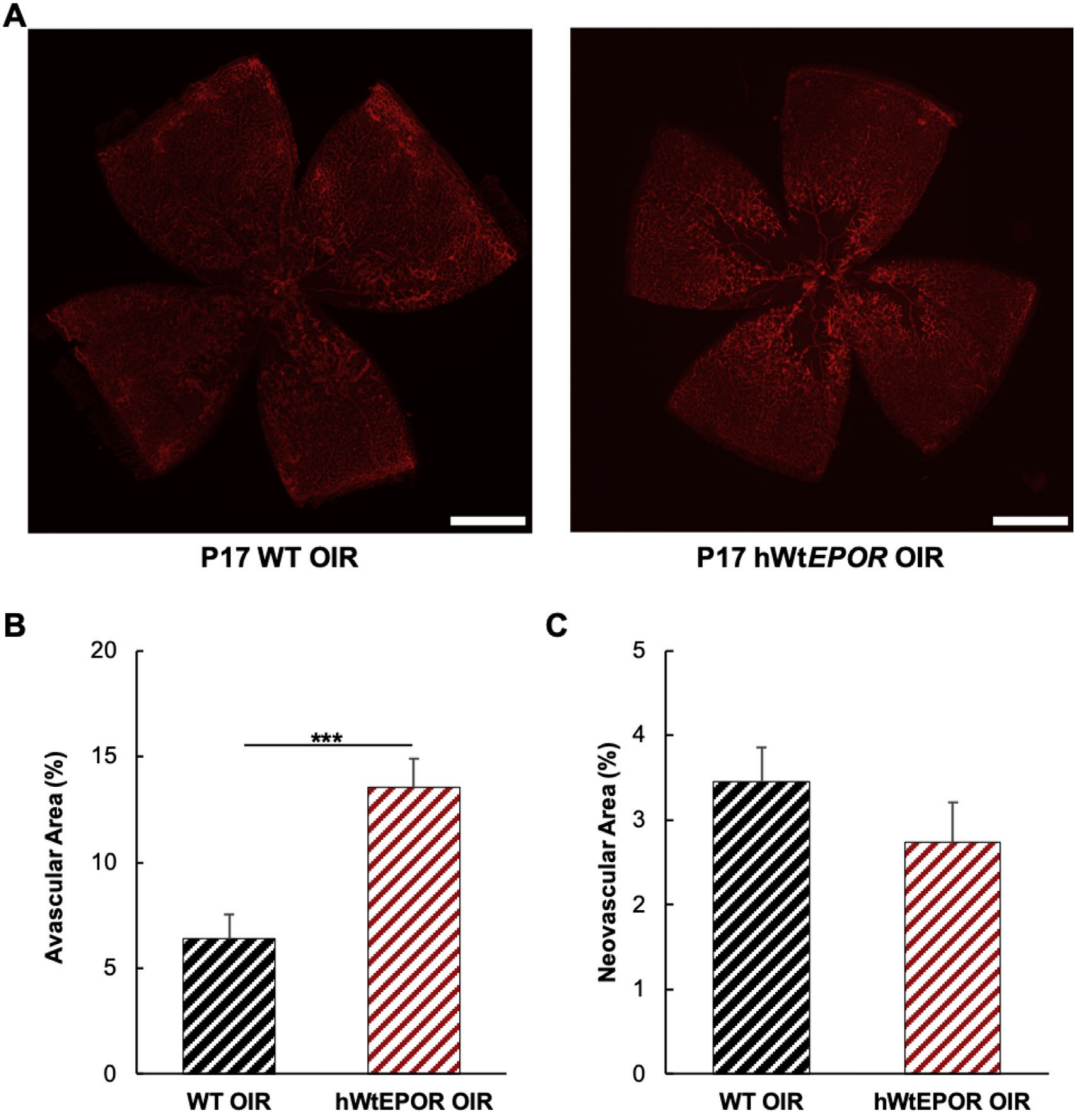
Effect of reduced EPOR signaling at p17 following OIR. (**A)** Representative images of p17 retinal flatmounts from WT and hWt*EPOR* mice. (**B)** Comparison of avascular area at p17 in WT and hWt*EPOR* mice (WT (n = 18), hWt*EPOR* (n = 13)). (**C)** Comparison of intravitreal neovascular area at p17 in WT and hWt*EPOR* mice (WT (n = 18), hWt*EPOR* (n = 13)). Results are means ±SEM. Scale bar = 500 µmol/L. *** *P* < 0.001.

Our hypothesis predicted that hWt*EPOR* mice would have reduced vascular regrowth after OIR and increased avascular area at p17. Indeed, at p17, hWt*EPOR* mice had significantly increased avascular retinal area compared with WT littermates (Fig. 4B), suggesting that reduced EPOR signaling impaired vascular regrowth after high oxygen. Intravitreal neovascularization (IVNV) after five days in hypoxia at p17 was not different between WT and hWt*EPOR* littermates (Fig. 4C) despite a significantly increased central avascular retina in the hWt*EPOR* mice. This finding supports the hypothesis that hypoactive EPOR signaling also reduced pathologic angiogenesis despite avascular retina, and potential hypoxia-induced, angiogenic effects.^27^ Altogether the data support our hypothesis that despite greater avascular retina and hypoxia-induced effects, hypoactive EPOR signaling resulted in reduced angiogenesis, seen as less vascular regrowth and less than expected IVNV.

### Expression of Angiogenic Factors

**Figure 5.**
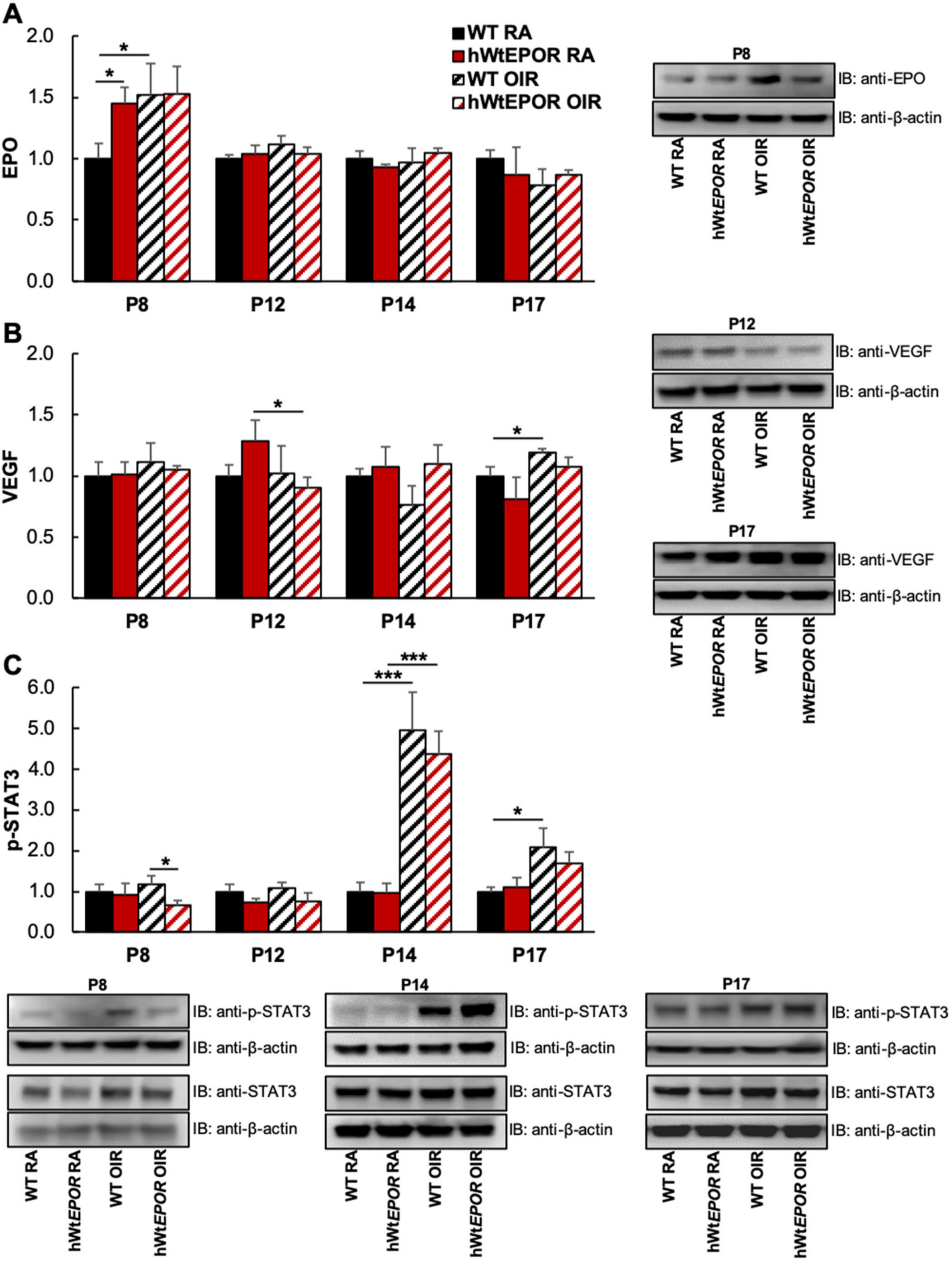
Effect of reduced EPOR signaling on angiogenic proteins in the retina. (**A)** Comparison of retinal Epo protein in RA and OIR treated mice at p8, p12, p14, and p17, with representative Western blot image from p8 (p8: WT RA (n = 6), hWt*EPOR* RA (n = 5), WT OIR (n = 6), hWt*EPOR* OIR (n = 5); p12: all groups (n = 6); p14: WT RA (n = 6), hWt*EPOR* RA (n = 5), WT OIR (n = 4), hWt*EPOR* OIR (n = 8); p17: WT RA (n = 6), hWt*EPOR* RA (n = 5), WT OIR (n = 6), hWt*EPOR* OIR (n = 6). (**B)** Comparison of retinal Vegf protein in RA and OIR treated mice at p8, p12, p14, and p17, with representative Western blot image from p17 (p8: WT RA (n = 6), hWt*EPOR* RA (n = 5), WT OIR (n = 6), hWt*EPOR* OIR (n = 5); p12: all groups (n = 6); p14: WT RA (n = 6), hWt*EPOR* RA (n = 5), WT OIR (n = 4), hWt*EPOR* OIR (n = 8); p17: WT RA (n = 6), hWt*EPOR* RA (n = 5), WT OIR (n = 6), hWt*EPOR* OIR (n = 6)). (**C)** Comparison of retinal p-Stat3 protein in RA and OIR treated mice at p8, p12, p14, and p17, with representative Western blot images from p8, p14, and p17 (p8: WT RA (n = 10), hWt*EPOR* RA (n = 9), WT OIR (n = 10), hWt*EPOR* OIR (n = 9); p12: all groups (n = 6); p14: WT RA (n = 6), hWt*EPOR* RA (n = 5), WT OIR (n = 4), hWt*EPOR* OIR (n = 8); p17: WT RA (n = 10), hWt*EPOR* RA (n = 9), WT OIR (n = 10), hWt*EPOR* OIR (n = 10)). Results are means ± SEM. *P < 0.05 and **P < 0.01.

A long-standing notion is that in ROP, retinal hypoxia from avascular retina triggers the expression of angiogenic factors, mainly VEGF, to cause intravitreal neovascularization once an infant is removed from high supplemental oxygen. However, we found that the larger areas of avascular retina in the hWt*EPOR* mice were not associated with greater IVNV, as would have been expected. Since EPO has been shown to regulate other angiogenic factors and angiogenesis,^28^ we postulated that reduced EPOR signaling might affect angiogenic factor expression and lead to reduced levels. Therefore we tested the prediction that the similarity in IVNV despite greater avascular retina in hWt*EPOR* compared with WT mice at p17 after OIR was due to reduced angiogenic signaling in hWt*EPOR* compared to WT retinas as detected by reduced expression levels or protein concentrations in VEGF or other angiogenic factors. To address this prediction, we measured whole retinal lysates from WT and hWt*EPOR* mice at p8, p12, p14, and p17 in RA and OIR mice for angiogenic factors, Epo and VEGF (Figs. 5A, 5B), and the downstream signaling effector p-Stat3 (Fig. 5C).^29^

At p8, Epo was greater in hWt*EPOR* retinas compared with WT retinas from mice raised in RA (Fig. 5A). Hyperoxia increased retinal Epo at p8 in WT mice compared with RA but did not further increase the already elevated retinal levels in RA-raised hWt*EPOR* mice. At p8, there was no effect on total Stat3 (Supplementary Fig. S2); however, p-Stat3 was reduced in hyperoxia-exposed hWt*EPOR* mice compared with WT littermates (Fig. 5C).

**Figure 6.**
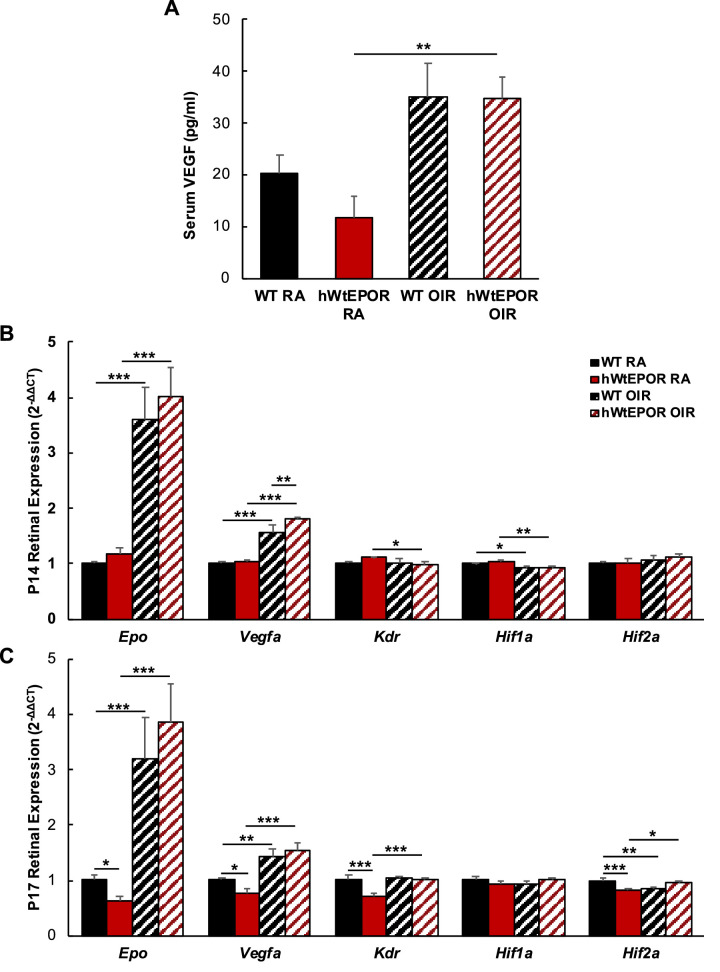
Effect of reduced EPOR signaling on angiogenic expression at p17. (**A)** Comparison of serum Vegf levels in RA and OIR WT and hWt*EPOR* mice at p17 (WT RA (n = 4), hWt*EPOR* RA (n = 4), WT OIR (n = 9), hWt*EPOR* OIR (n = 6)). (**B,** **C)** Comparison of retinal gene expression levels in RA and OIR WT and hWt*EPOR* mice at (**B)** p14 and (**C)** p17 (p14: WT RA (n = 6), hWt*EPOR* RA (n = 5), WT OIR (n = 4), hWt*EPOR* OIR (n = 8); p17: WT RA (n = 8), hWt*EPOR* RA (n = 6), WT OIR (n = 7), hWt*EPOR* OIR (n = 6)). Results are means ±SEM. * *P* < 0.05, ** *P* < 0.01, and *** *P* < 0.001.

At p12, p14, and p17, there were no differences in retinal Epo between RA and OIR treated mice of either genotype (Fig. 5A). Analyses at p8, p12, and p14 of retinal VEGF were also not significantly different between genotypes in RA or OIR (Fig. 5B). As predicted, WT mice had increased VEGF at p17 after OIR (Fig. 5B) but did not have significantly different protein levels from hWt*EPOR* littermates. Also, p-Stat3 was significantly increased in both genotypes in OIR compared with respective RA retinas at p14 but was not different between WT and hWt*EPOR* retinas (Fig. 5C). There was also no difference in serum VEGF between WT and hWt*EPOR* OIR mice at p17 (Fig. 6A), although hWt*EPOR* OIR mice had higher serum VEGF levels compared with RA hWt*EPOR* mice.

We also measured expression levels of mRNA for *Epo*, *Vegfa*, *Kdr*, *Hif1α*, and *Hif2α* between genotypes in RA and OIR at p14 and p17 (Figs. 6B, 6C). At p14 RA baseline retinal expression levels were no different between genotypes, but at p17 *Epo*, *Vegfa*, *Kdr*, and *Hif2α* were reduced in RA hWt*EPOR* retinas compared to littermate WT. At both p14 and p17, OIR led to increased retinal mRNA expression levels of *Epo* and *Vegfa* in both WT and hWt*EPOR* mice. However, in p14 OIR, hWt*EPOR* retinas had increased *Vegfa* expression compared with littermate WT, contrary to our prediction. *Hif1α* expression was reduced by OIR in both WT and hWt*EPOR* mice at p14 but unchanged at p17. *Hif2α* expression was unchanged at p14, but at p17 was reduced by OIR treatment in WT mice compared with WT RA, and increased in hWt*EPOR* OIR mice compared with hWt*EPOR* RA. The lack of significant increases in expression in WT compared with hWt*EPOR* after OIR refutes the prediction that hypoxia induced angiogenic factor expression, and proposed signaling, account for the similar area of IVNV despite greater avascular area in hWt*EPOR* retinas after OIR. Instead, the findings support the notion that EPOR Signaling was involved in angiogenesis regardless of avascular retina.

## Discussion

**Figure 7.**
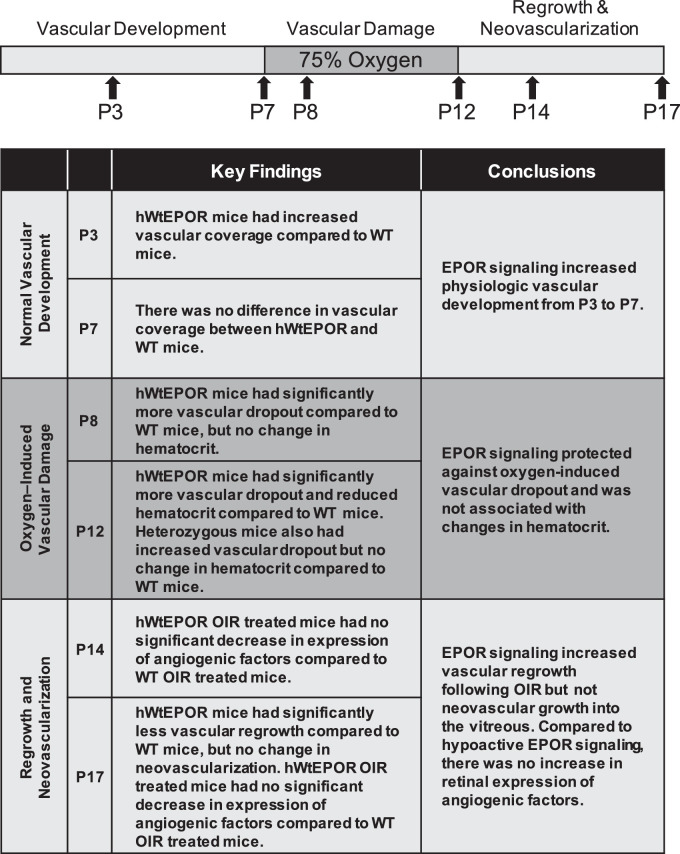
Oxygen-induced retinopathy model and key findings at timepoints analyzed. Mouse pups were raised in room air conditions until P7. Retinal vascular coverage was measured in P3 and P7 pups. At P7, some litters were placed in 75% oxygen for five days to induce vascular dropout, assessed as central avascular area in P8 and P12 pups. On P12, pups were returned to room air for five days. At p17, vascular regrowth, assessed by avascular area, and intravitreal neovascularization were measured. Retinal protein levels of Epo, VEGF, and p-Stat3 were measured at P8, P12, P14, and P17 and retinal mRNA expression of angiogenic factors *Epo*, *Vegfa*, *Kdr*, *Hif1α*, and *Hif2α* were measured at p14 and p17.

We found evidence that EPOR signaling was important in not only stabilizing high oxygen exposed newly developed capillaries but also in supporting regrowth of vasculature into damaged retinal beds and of intravitreal vascular growth (Fig. 7). We provided evidence using a unique transgenic humanized model in which hypoactive EPOR signaling led to increased hyperoxia-induced central avascular retina without induction of anticipated relative hypoxia-induced IVNV. Furthermore, the model provides a means to control for EPOR signaling even when EPO is increased. Our study provides further evidence that EPOR signaling plays a role in angiogenesis even when EPO, as a ligand, is able to interact with other receptors proposed to cause tissue protection or angiogenesis.^16^ Our findings also provide some insight into the effect of EPOR signaling on angiogenesis related to previous experimental studies using exogenous EPO or knockdown of EPO.^1^^,^^4^

Although the mouse OIR model does not represent all aspects of ROP, it reflects oxygen-induced changes to newly formed capillaries, which can occur with insufficient resources to regulate and monitor oxygen.^30^^,^^31^ It also is an excellent model to test hypoxia-induced angiogenesis. The model is also useful to study retinovascular diseases that have loss of capillary support followed by intravitreal growth of vessels, such as diabetic retinopathy and branch retinal vein occlusion.

An important aspect is that hypoactive EPOR signaling occurs throughout organ systems of the animal, including the bone marrow, thereby affecting hematocrit. However, we found lack of associations between hematocrit and avascular retina at p8 and at p12 in heterozygous mice, providing support for our hypothesis that the effect of EPOR signaling was through angiogenesis. At p17, hematocrits were reduced in the hWt*EPOR* mice but given the expected associated reduction in oxygen carrying capacity of the blood and increased avascular area, hypoxia induced IVNV was not increased compared to WT. This also supports the role of EPOR signaling in angiogenesis.

Hypoxia-induced expression of angiogenic factors, including VEGF, has been reported in a number of retinal cells,^32^^–^^34^ including Müller cells, RPE, astrocytes, and less often in endothelial cells, which have receptors that are activated by secreted angiogenic factors to result in angiogenesis. The larger areas of avascular retina at p12 and p17 in the hWt*EPOR* compared with WT mice would have been expected to lead to greater IVNV if hypoxia-induced expression of angiogenic factors occurred in retinal cells. However, this was not found. IVNV was similar between genotypes, as was the expression levels of angiogenic factors tested. The cause for this is unclear. In many studies, EPOR induces angiogenic factor expression but EPOR signaling also interacts with VEGF signaling, in which angiogenesis is potentiated by these two signaling pathways.^28^ In certain cancers, such as multiple myeloma, recombinant EPO was shown to downregulate angiogenic factors.^35^ Part of the effect in this study may be due to the uniqueness of the animal model affecting EPOR signaling throughout the entire mouse that may have compensatory signaling effects in other cell types that then affect signaling within the vasculature of the retina. Future studies to consider the effects of EPOR signaling specifically in retinal endothelial cells will also be considered as will the identification of potential angiogenic inhibitors.

Unexpectedly, hypoactive EPOR signaling appeared associated with more retinal vascularization at p3. Increased exogenous EPO has been shown to increase the number of retinal microglia and endothelial progenitor cells associated with developing retinal vasculature.^1^ However, increased circulating Epo with hypoactive EPOR signaling is a unique feature of our model, and one possibility is that the effects on other cell types are not mediated through EPO/EPOR signaling but rather through receptors other than EPOR. More study is needed to assess this finding at other time points and to determine a potential mechanism. We found that vascular repair in OIR at p17 was also impaired in the hWt*EPOR* mice compared with WT mice. This finding aligns with that of previous investigations that found EPO supported vascular stability from OIR.^1^

Our study addresses the effects of EPOR signaling on pathologic and physiological angiogenesis without affecting other receptors that EPO activates. It does not address the question whether exogenous EPO is beneficial in retinal diseases. Clinical studies show evidence of a beneficial association between increased endogenous EPO and reduced diabetic retinopathy in humans.^36^ The PENUT study failed to show effects of early administration of exogenous EPO in premature infants born less than 28 weeks gestation on ROP or on neurocognitive milestones at age 2, which was different from a meta-analysis of four randomized studies that found EPO reduced the risk neurocognitive dysfunction. Enrollment in the PENUT, however, excluded infants at high risk of conditions that can affect neurodevelopment and ROP, including low hematocrit.^37^ It will also be important to test the cohort at older ages when neurocognitive testing is more reliable.

In conclusion, our study provides evidence that EPOR signaling is involved in retinal angiogenesis, whether it be reparative following oxygen-induced capillary dropout or following hypoxia-induced intravitreal angiogenesis, even when EPO, as a ligand, is available to trigger signaling through other receptors. Our study also suggests that in situations of reduced EPOR signaling, angiogenesis whether physiological or pathologic is reduced after oxygen stresses.

## Supplementary Material

Supplement 1

Supplement 2
