# Supplementary material for: Signaling Through the Erythropoietin Receptor Affects Angiogenesis in Retinovascular Disease
Source: Invest Ophthalmol Vis Sci. 2020 Aug 12;61(10):23. doi: 10.1167/iovs.61.10.23 (PMC7441364; doi:10.1167/iovs.61.10.23)
Supplement: Supplement 1 [file iovs-61-10-23_s001.pdf]

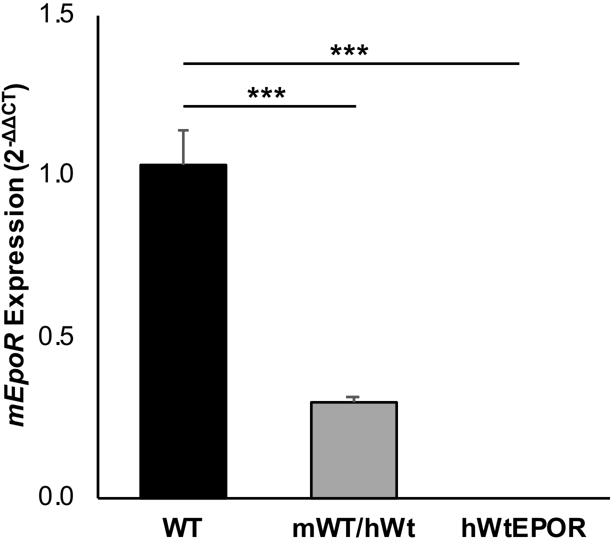

1.5
1.0
0.5
0.0
mEpoR Expression (2^-ΔΔCT)
***
***
WT
mWT/hWt
hWtEPOR

Supplemental Figure 1. Retinal murine *EpoR* expression.

Comparison of murine *EpoR* expression in retinas of p17 WT, heterozygous, and hWt*EPOR* mice (WT (n=8), heterozygous (n=3), hWt*EPOR* (n=3)). Results are means ±SEM. *** = $p<0.001$
