# Supplementary material for: Signaling Through the Erythropoietin Receptor Affects Angiogenesis in Retinovascular Disease
Source: Invest Ophthalmol Vis Sci. 2020 Aug 12;61(10):23. doi: 10.1167/iovs.61.10.23 (PMC7441364; doi:10.1167/iovs.61.10.23)
Supplement: Supplement 2 [file iovs-61-10-23_s002.pdf]

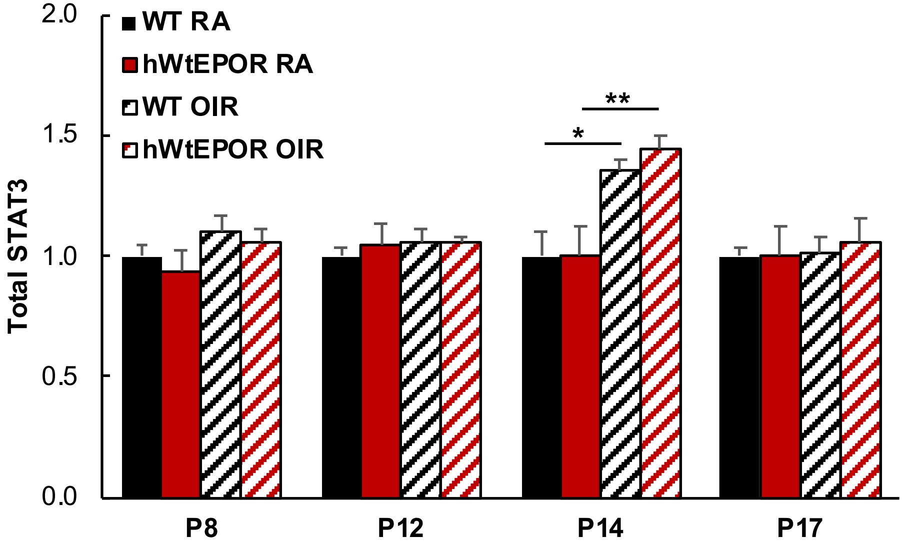

WT RA
hWtEPOR RA
WT OIR
hWtEPOR OIR
Total STAT3
2.0
1.5
1.0
0.5
0.0
*
**
P8
P12
P14
P17

Supplemental Figure 2. Retinal levels of total STAT3.

Comparison of retinal total Stat3 in RA and OIR WT and hWt*EPOR* mice at p8, p12, p14, and p17 (p8: all groups (n=4); p12: all groups (n=3); p14: WT RA (n=6), hWt*EPOR* RA (n=5), WT OIR (n=4), hWt*EPOR* OIR (n=8); p17: WT RA (n=6), hWt*EPOR* RA (n=5), WT OIR (n=6), hWt*EPOR* OIR (n=6)). Results are means ±SEM. * = $p<0.05$ and ** = $p<0.01$
